# Predicting Freezing of Gait in Parkinson’s Disease: A Machine Learning-Based Approach in ON and OFF Medication States

**DOI:** 10.3390/jcm14062120

**Published:** 2025-03-20

**Authors:** Georgios Bouchouras, Georgios Sofianidis, Konstantinos Kotis

**Affiliations:** 1 Rehabilitation, School of Health Sciences, Metropolitan College, 54624 Thessaloniki, Greece; gsofianidis@mitropolitiko.edu.gr; 2 Intelligent Systems Lab, Department of Cultural Technology and Communication, University of the Aegean, 81100 Mytilene, Greece; kotis@aegean.gr

**Keywords:** freezing of gait (FoG), Parkinson’s disease (PD), machine learning (ML), gait analysis, random forest regressor

## Abstract

**Background**: Freezing of gait (FoG) is a debilitating motor symptom of Parkinson’s disease (PD), characterized by sudden episodes where patients struggle to initiate or sustain movement, often describing a sensation of their feet being “glued to the ground.” This study investigates the potential of machine-learning (ML) models to predict FoG severity in PD patients, focusing on the influence of dopaminergic medication by comparing gait parameters in ON and OFF medication states. **Methods**: Specifically, this study employed spatiotemporal gait features to develop a predictive model for FoG severity, leveraging a random forest regressor to identify the most influential gait parameters associated with this in each medication state. The results indicate that the model achieved higher predictive performance in the OFF-medication condition (R² = 0.82, MAE = 2.25, MSE = 15.23) compared to the ON-medication condition (R² = 0.52, MAE = 4.16, MSE = 42.00). **Results**: These findings suggest that dopaminergic treatment alters gait dynamics, potentially reducing the reliability of FoG predictions when patients are medicated. Feature importance analysis revealed distinct gait characteristics associated with FoG severity across medication states. In the OFF condition, step length parameters, particularly left step length mean, were the most dominant predictors, alongside swing time and stride width, indicating the role of spatial and temporal gait control in FoG severity without medication. In contrast, under the ON medication condition, stride width and gait speed emerged as the most influential predictors, followed by stepping frequency, reflecting how medication influences stability and movement rhythm. **Conclusions**: These findings highlight the need for predictive models that account for medication-induced gait variability, ensuring more reliable FoG detection. By integrating spatiotemporal gait analysis and ML-based prediction, this study contributes to the development of personalized intervention strategies for PD patients experiencing FoG episodes.

## 1. Introduction

Freezing of gait (FoG) is a prevalent and debilitating symptom of Parkinson’s disease (PD), characterized by sudden episodes in which patients are unable to initiate or sustain movement, often feeling as if their feet are `glued to the ground’ [1,2]. These episodes significantly impair mobility and increase the risk of falls, making FoG a major challenge in PD management. The underlying pathophysiology of FoG is complex, involving disruptions in neural circuits responsible for gait control, particularly within the basal ganglia and supplementary motor areas [3,4]. While dopaminergic therapies, such as levodopa, alleviate many PD motor symptoms, their effectiveness in treating FoG is inconsistent, a phenomenon known as the “levodopa paradox” [1,5]. This inconsistency highlights the need for alternative approaches to predict and manage FoG, particularly by analyzing spatiotemporal gait parameters that capture subtle gait disturbances.

Recent advances in machine learning (ML) have provided powerful tools for analyzing complex gait datasets, allowing researchers to detect patterns that may not be evident through traditional statistical approaches [6,7]. By integrating spatiotemporal gait data collected from wearable sensors, predictive models can assess gait dynamics across different states of medications, providing deeper insight into how medication modulates gait and contributes to FoG episodes [6,8]. This study explores the potential of ML-based approaches to predict the severity of FoG in PD patients, focusing on the differences in gait parameters between the ON and OFF medication states. The findings may contribute to the development of more precise and individualized rehabilitation strategies for PD patients experiencing FoG.

Understanding the impact of medication on gait variability is essential to improve the prediction and management of FoG. The primary aim of this study was to predict FoG using spatiotemporal gait parameters and assess the effectiveness of gait-based models in identifying FoG episodes in individuals with PD. Additionally, the study sought to investigate whether the predictive performance of FoG differs between ON and OFF medication conditions, evaluating the influence of dopaminergic treatment on gait-related FoG prediction. A further objective was to examine how spatiotemporal gait variations contribute to FoG prediction, determining whether specific gait characteristics hold different predictive relevance depending on medication status.

The structure of this paper is as follows: Section 2 presents a review of related work, providing an overview of previous studies on FoG, the impact of dopaminergic treatment on gait, and advancements in machine-learning-based prediction models. Section 3 outlines the research methodology, including data collection, preprocessing, and the application of a random forest regressor to analyze spatiotemporal gait parameters. Section 4 details the results, comparing the model’s predictive performance across ON and OFF medication states, with an emphasis on the influence of specific gait parameters. In Section 5, the discussion interprets the findings in the context of the levodopa paradox and examines the clinical implications of medication-induced gait variability. Lastly, Section 6 concludes the study by summarizing key insights, highlighting limitations, and suggesting directions for future research to enhance FoG prediction and intervention strategies in PD.

## 2. Related Work

FoG is a complex and debilitating symptom of PD, characterized by intermittent episodes in which patients experience an inability to initiate or maintain movement. Research has extensively documented the spatiotemporal gait parameters associated with FoG and the effects of dopaminergic medications on these parameters. For instance, studies have shown that dopaminergic therapy can lead to significant changes in gait dynamics, with variations in stride length, swing time, and step width being particularly pronounced in patients with a history of FoG [1,9]. The “levodopa paradox” illustrates that while dopaminergic medications can alleviate many motor symptoms, they often fail to adequately address FoG, leading to a need for alternative predictive models that can account for these inconsistencies [10,11].

Recent advances in ML and wearable technology have opened new avenues for predicting FoG episodes by analyzing gait characteristics in real-time. For example, Borzi et al. (2021) demonstrated the potential of wearable sensors to capture gait patterns preceding FoG episodes, allowing for timely interventions [6]. Similarly, studies have highlighted the effectiveness of various cue strategies, such as tactile and auditory cues, in mitigating symptoms of FoG during gait tasks [12,13,14]. These interventions leverage the understanding that external cues can facilitate movement initiation and improve gait performance, particularly in challenging situations such as turning or navigating obstacles [15,16,17].

The neural mechanisms underlying FoG have also been a focal point of research. Investigations into the cholinergic system have revealed that cholinergic degeneration correlates with gait impairments in PD, suggesting that cognitive factors may exacerbate gait disturbances [18,19]. Furthermore, neuroimaging studies have provided insights into the structural and functional changes in brain networks associated with FoG, indicating that disruptions in the basal ganglia and supplementary motor areas play a critical role in the manifestation of this symptom [3,4,20]. These findings highlight the multifaceted nature of FoG, which is influenced by both motor and cognitive domains.

Furthermore, the impact of dual tasks on gait performance has been extensively studied, revealing that cognitive load can significantly impair gait stability and increase the probability of FoG episodes [21]. For instance, Spildooren et al. (2010) noted that individuals with FoG exhibited greater gait asymmetry and variability when engaged in dual-task conditions, highlighting the importance of attentional resources in maintaining gait control [15]. This aligns with the findings of Shine et al. (2013), which indicated that concurrent cognitive tasks can alter neural activation patterns, further complicating FoG treatment in patients with PD [22].

Emerging research on postural control has provided further insights into mobility impairments in PD. Mangalam et al. (2024) examined the directional characteristics of postural sway in young adults, older adults, and individuals with PD, utilizing oriented fractal scaling component analysis [23]. Their findings revealed that while healthy young adults control posture along traditional anteroposterior and mediolateral axes, older adults and individuals with PD exhibit sway along suborthogonal directions that significantly deviate from these axes. This deviation suggests altered temporal structures of sway variability, indicative of postural deficits that extend beyond age-related changes alone. Notably, individuals with PD displayed a threefold increase in sway variability compared to healthy older adults, underscoring the profound impact of neurodegeneration on postural control.

The integration of wearable sensors and ML techniques has also been explored for predicting fall risk among PD patients, as FoG episodes are a major contributor to falls. Given that FoG is often accompanied by sudden movement cessation and postural instability, identifying gait patterns associated with these episodes can provide crucial insights into fall susceptibility. By leveraging ML-based models to analyze gait variability and postural sway, researchers aim to enhance fall risk assessment and develop targeted interventions to mitigate the likelihood of falls in individuals with PD. Sotirakis et al. (2024) conducted a five-year longitudinal study assessing 104 PD participants using wearable sensors during walking and postural sway examinations. Their findings indicated that walking and postural variability are significant predictors of future falls, with a random forest classifier achieving an accuracy of 78% at 60 months. Incorporating demographic factors, particularly age, further improved the predictive performance of their model, highlighting the potential of objective kinematic data in enhancing fall risk assessment and management strategies [24].

Postural sway variability has also been studied as a biomarker for PD progression. Kiyono, Mangalam, and Stergiou (2024) found that people with PD exhibit a highly deterministic structure in their postural sway, leading to more rigid and predictable behaviors compared to their healthy counterparts. Their research suggests that patients with PD show a pronounced insensitivity to task-related changes in postural stability, indicating reliance on inflexible control strategies. These findings reinforce the importance of postural control in PD diagnostics and patient management [23].

Recent advancements in artificial intelligence have greatly enhanced the understanding of PD-related motor symptoms, such as bradykinesia and tremor, which are closely associated with gait disturbances and the onset of FoG. To deepen domain-specific knowledge, researchers have leveraged ontology-based methodologies and large language models (LLMs) to systematically characterize these symptoms and their effects on movement. By structuring and integrating clinical and biomechanical data, these approaches improve the prediction and monitoring of FoG episodes, particularly in relation to medication states and individual patient responses. Bouchouras et al. (2024) introduced X-HCOME, a hybrid ontology engineering methodology that combines the computational capabilities of LLM with human expertise to enhance the efficiency and precision of ontology generation for PD monitoring. Their study focused on detecting bradykinesia and tremor, two hallmark motor impairments of the disease, demonstrating that LLM-assisted ontology development significantly improves class generation and predictive performance [25]. Similarly, Doumanas et al. (2025) explored the role of LLMs in collaborative ontology engineering, highlighting that while LLMs expedite ontology construction, human expertise remains essential for maintaining logical consistency and domain-specific precision [26]. Building on these advancements, ontology-driven methodologies can play a crucial role in predicting FoG in PD by structuring gait-related features for ML-based models. Since bradykinesia and tremor—two impairments studied by Bouchouras et al. (2024) [25]—are closely linked to FoG, their ontology-based classification can enhance feature selection and model interpretability. By integrating LLM-assisted ontology generation, ML models can better capture clinically relevant gait alterations, ensuring a more precise and context-aware FoG prediction framework.

In summary, integration of ML techniques with spatiotemporal gait analysis presents a promising approach to predict the severity of FoG in PD. By understanding the complex interaction between medication status, cognitive load, and gait parameters, researchers can develop more effective predictive models and interventions aimed at improving mobility and reducing the risk of falls in individuals with PD. Although previous studies have examined the effects of dopaminergic medication, wearable sensors, and cue strategies on FoG, there is limited research on how spatiotemporal gait features contribute to FoG prediction in ON and OFF medication states. Existing models often do not differentiate between these conditions, potentially overlooking medication-induced gait variability. By applying ML to analyze spatiotemporal gait characteristics separately for the ON and OFF states, this study explores whether different gait features contribute differently to FoG prediction in each condition. Understanding these differences may provide insights into the role of medication in gait mechanics and help refine predictive models for more targeted intervention strategies.

## 3. Research Methodology

### 3.1. Data Collection

This study utilizes a publicly available dataset collected by Shida et al. (2023) [Dataset Link (https://www.frontiersin.org/journals/neuroscience/articles/10.3389/fnins.2023.992585/full#supplementary-material), accessed on 17 March 2025], which provides full-body kinematics and kinetics of individuals with PD [27].

The dataset includes 26 PD patients, aged between 44 and 81 years (Table 1), with varying disease severity classified using the Hoehn and Yahr scale (stages 1–4). Among them, 13 participants exhibited FoG. Participants were on a stable dose of L-DOPA, and gait data were recorded in two conditions: **ON** and **OFF** medication.

The study was carried out at the Laboratory of Biomechanics and Motor Control of the Federal University of ABC, Brazil, with ethical approval from the local Ethics Committee (protocol number 21948619.6.0000.5594, 4 November 2019).

Comprehensive clinical assessments were performed to evaluate motor and cognitive function, including the Unified PD Rating Scale (**UPDRS-II & III**), New FoG Questionnaire (**NFoG-Q**), Mini-BESTest, and Fall Efficacy Scale (**FES-I**), among others. These assessments ensured a detailed characterization of gait dysfunction in the participants, providing valuable clinical context to the collected gait data.

**Figure 1:**
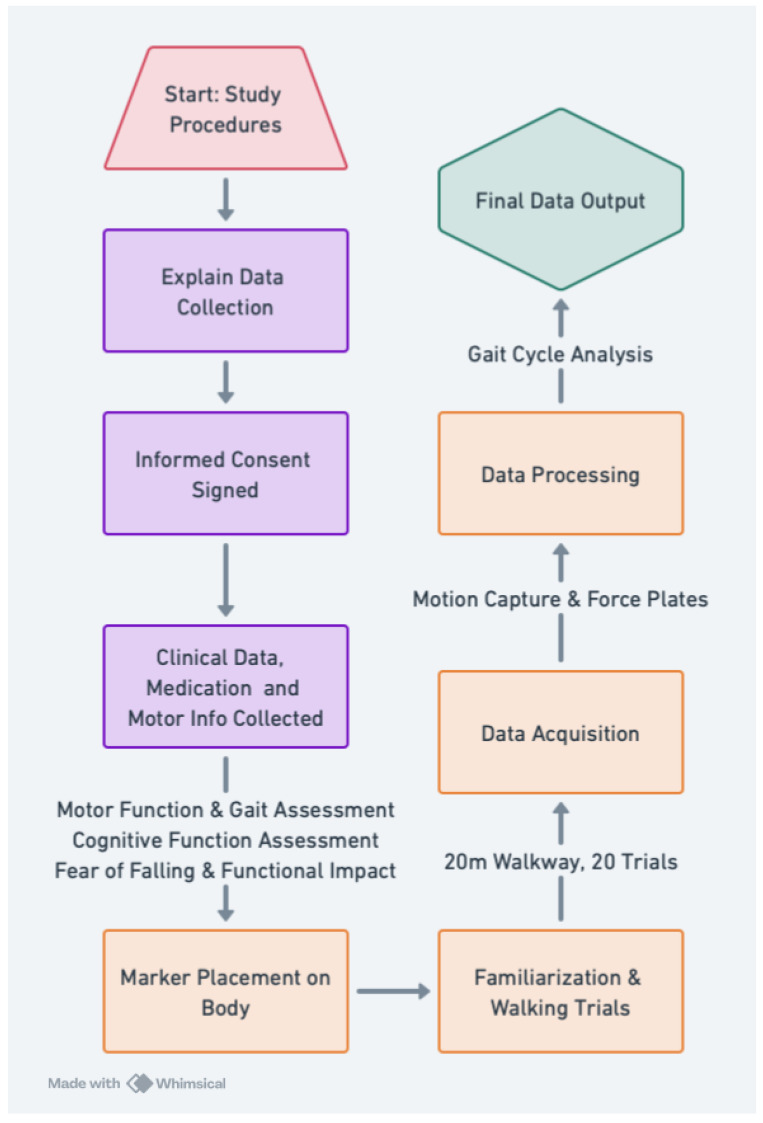
Flowchart depicting the data collection and processing workflow for gait analysis. The study procedures include participant consent, clinical assessments, motion capture setup, and data acquisition. The data were collected by Shida et al (2023) [27]. The flowchart was created using Whimsical, Last access date: 15 March 2025 https://whimsical.com/.

Gait data collection involved barefoot overground walking trials along a 20-m walkway at a self-selected comfortable speed (Figure 1). Each participant completed 20 walking trials while being recorded using a 12-camera motion capture system (Raptor-4, Motion Analysis Corp., Rohnert Park, CA, USA) at 150 Hz. Ground reaction forces were measured using five embedded force plates, sampled at 300 Hz. A 44-marker set was placed on anatomical landmarks based on the guidelines of the International Society of Biomechanics, and a T-pose calibration trial was conducted for the baseline assessment of posture. Heel-strike and toe-off events were automatically detected using a marker velocity-based algorithm to ensure accurate gait cycle segmentation.

The study involved participants walking in both ON and OFF medication conditions to assess the impact of dopaminergic medication on gait. *ON Medication Condition*: Participants took their dopaminergic medication 1 h before the session to ensure that medication levels were stable. Participants were required to be on a stable dose of L-DOPA for at least one month prior to the study. The mean L-Dopa equivalent dose for the participants was reported as 845.65 mg/day. *OFF Medication Condition*: Participants refrained from taking their medication for at least 12 h before the experimental session, allowing the researchers to observe the effects of having no medication on gait performance. The dataset includes raw C3D and ASCII files containing 3D marker trajectories and force plate data, as well as processed kinematic and kinetic time-normalized curves across 101 time points per gait cycle. Data were interpolated using a cubic spline algorithm and analyzed in Visual3D (C-Motion, Inc., Germantown, MD, USA). The dataset provides detailed spatiotemporal parameters, including stride length, step duration, and cadence, allowing for a comprehensive analysis of gait mechanics in PD. This dataset offers high-resolution gait kinematics and kinetics, enabling the development of ML models for gait anomaly detection. The inclusion of both ON and OFF medication states allows for a more robust evaluation of gait variability in PD, making it a valuable resource for deep-learning applications in clinical gait analysis.

The current study specifically focuses on the spatiotemporal gait characteristics, medication statue, and FoG scores of the participants, as these variables align with our research objective of predicting FoG in PD across ON and OFF medication states. Although the data set collected by Shida et al. (2023) [27] provides a comprehensive range of kinematic and kinetic parameters, including full-body motion capture data and ground reaction forces, UPDRS scores, Hoehn and Yahr stage, and other clinical assessments, we deliberately selected only spatiotemporal characteristics and FoG scores for analysis. This choice ensures that our model remains targeted towards gait-related predictors of FoG without introducing additional biomechanical complexity that falls outside the scope of our study. However, we present the full data collection methodology to provide readers with a clear understanding of the richness of the data set, facilitating transparency and reproducibility. By outlining the broader data acquisition process, we allow future studies to explore additional aspects of gait analysis while reinforcing the rationale for our variable selection in the current investigation. Finally, in the current study, 4 of the 26 initially included participants were excluded due to incomplete/missing gait cycle data, leaving a final analyzed sample of 22 participants. The clinical outcome this study used was the FoG score.

### 3.2. Data Prepossessing

The data used for this analysis comprised two primary sources: spatiotemporal gait cycle files and clinical FoG scores. The gait cycle files were stored in a specified directory, while the FoG scores were obtained from a separate clinical dataset. Data preprocessing and analysis were conducted using Python, version 3.13.2, with the Pandas library for data handling, and scikit-learn for ML modeling.

The initial step involved loading the FoG score data. This dataset contained patient IDs and corresponding FoG-Q scores, which quantify the severity of FoG. To ensure consistency during the merging process, the patient IDs were treated as strings. Following this, the spatiotemporal gait data was read from individual files. Each file contained gait parameters for a specific patient, with filenames indicating the patient ID and their medication state (ON or OFF). The relevant spatiotemporal parameters, including stride length, swing time, and step width, were extracted and organized into a structured DataFrame. Each patient’s data was annotated with their medication status, which was converted to a numerical format for analysis (ON = 1, OFF = 0).

After loading the data, the spatiotemporal gait features were merged with the FoG scores using the patient ID as a common key. This integration allowed for a comprehensive dataset linking gait characteristics to FoG severity. A check for missing FoG-Q scores was performed, but no missing values were found. The dataset was processed to ensure data integrity. The resulting dataset was then partitioned into two subsets based on medication status: ON and OFF. This stratification was essential for understanding how medication influences gait

### 3.3. Random Forest Regressor

For the predictive modeling, a random forest regressor was employed due to its ability to handle non-linear relationships and its robustness against overfitting. The random forest regressor was chosen for its robustness in handling nonlinear relationships and its ability to provide interpretable feature rankings, making it an effective tool for FoG prediction in PD patients [4,5]. The model used in this study is an ensemble learning model that constructs multiple decision trees and averages their predictions to enhance accuracy and reduce overfitting. The model was configured with 100 trees, using bootstrap sampling (bootstrap=True) to introduce variability and improve generalization. Each tree was grown without a predefined depth limit, allowing for flexible decision boundaries, while nodes were split based on mean squared error. To ensure reproducibility, a fixed random seed was set, and the model utilized all available CPU cores for computational efficiency. Before model training, spatiotemporal features were standardized using z-score normalization to ensure comparability between variables with different scales. The standardized data were split into training and testing sets, with 80% of the data used for model training and 20% reserved for testing. This approach facilitated the evaluation of the generalizability of the model to unseen data. The random forest model was trained separately for the ON and OFF medication datasets, allowing for a focused analysis of how gait parameters contribute to FoG prediction under different conditions. During training, the model learned to identify patterns in the spatiotemporal features that correlated with FoG-Q scores. After training, the model performance was evaluated using standard regression metrics. Mean absolute error (MAE), mean squared error (MSE), and R2 score, which shows how much of the variation in the dependent variable is explained by the independent variables. These metrics provided a quantitative measure of the accuracy of the model in predicting the severity of FoG, with lower MAE and MSE indicating better performance, and higher R² values reflecting greater explanatory power.

### 3.4. Feature Important Analysis and Pairwise Comparisons

To identify the most influential spatiotemporal gait parameters contributing to FoG severity prediction, two feature importance methods were applied: impurity-based importance and permutation importance. These methods provide complementary insights into how different gait parameters influence the model’s predictive performance.

The first method, impurity-based importance, is an intrinsic property of the random forest model. It measures how much each feature reduces uncertainty (impurity) in the model’s decision-making process. In random forest, decision trees split the dataset at various feature values to create more homogeneous groups. The more a feature helps in making these splits and reducing uncertainty, the higher its importance score. In this study, impurity-based importance was computed as the average decrease in impurity (e.g., variance in regression) across all trees in the forest. Features that contributed more to accurately predicting FoG severity received higher importance scores, indicating their relevance in distinguishing between different levels of FoG severity.

The second method, permutation importance, is a model-agnostic approach that evaluates how much a feature contributes to the model’s performance by randomly shuffling its values and measuring the resulting change in prediction accuracy. The process involves making predictions with the trained model using the original dataset and recording its performance. Then, the values of a single feature, such as stride length, are randomly shuffled, disrupting any meaningful relationship between that feature and the target variable. The model then makes new predictions with this shuffled dataset, and the change in performance is recorded. If the model’s predictive accuracy deteriorates significantly after shuffling a specific feature, it indicates that the feature was highly important for making accurate predictions.

By applying these feature importance methods separately for ON and OFF medication states, the study could determine which gait parameters were most predictive of FoG severity in each condition. This comparison also provided insights into how medication influences the predictive power of different gait parameters, helping understanding of its effects on motor control in PD patients. These analyses enabled the ranking of the most influential spatiotemporal features, offering valuable information about gait alterations related to FoG and the potential impact of medication on these patterns.

Furthermore, paired sample t-tests were performed for each spatiotemporal gait parameter between ON and OFF medication conditions, ensuring that comparisons account for within-subject variability. This statistical test aimed to determine whether there were significant differences in gait parameters within the same participants across medication states, providing additional information on how medication modulates gait patterns in PD patients. The significance level was set at *p* < 0.05.

The methodology presented in this study is supported by a publicly available GitHub repository: https://github.com/GiorgosBouh/PD_spatiotemporal_Regression (last accessed: 15 March 2025), which contains all the codes, datasets, and processing scripts used for the analysis. This ensures transparency, reproducibility, and accessibility for researchers aiming to validate or extend the findings. The repository includes preprocessed spatiotemporal gait data, FoG scores, and machine-learning models, enabling direct replication of the study’s approach. By providing full access to the computational framework, this work promotes open science practices and facilitates future advancements in FoG prediction and PD patients’ gait analysis. This ensures transparency, reproducibility, and accessibility for researchers aiming to validate or extend the findings. The repository includes preprocessed spatiotemporal gait data, FoG scores, and machine-learning models, enabling direct replication of the study’s approach. By providing full access to the computational framework, this work promotes open science practices and facilitates future advancements in FoG prediction and PD patients’ gait analysis.

## 4. Results

### 4.1. Model Performance Across ON and OFF Medication Conditions

The performance of the random forest regressor was evaluated separately for ON and OFF medication conditions using mean absolute error (MAE), Mean Squared Error (MSE), and the R² score. The results indicate that the model performed better in the OFF medication condition compared to the ON medication condition (Table 2).

For the ON medication condition, the model achieved an MAE of 4.16, indicating that the predicted FoG scores deviated from the actual values by an average of 4.16 points. The MSE was 42.00, suggesting larger prediction errors, and the R² score of 0.52 indicates that the model explains 52% of the variance in FoG scores under medication.

In contrast, for the OFF medication condition, the model demonstrated improved performance, with an MAE of 2.25, an MSE of 15.23, and an R² score of 0.82, meaning that 82% of the variance in FoG scores was explained, suggesting a stronger relationship between spatiotemporal gait parameters and FoG severity when patients were not under medication.

### 4.2. Feature Analysis

Feature Importance Analysis in ON vs. OFF Medication Conditions

**Figure 2:**
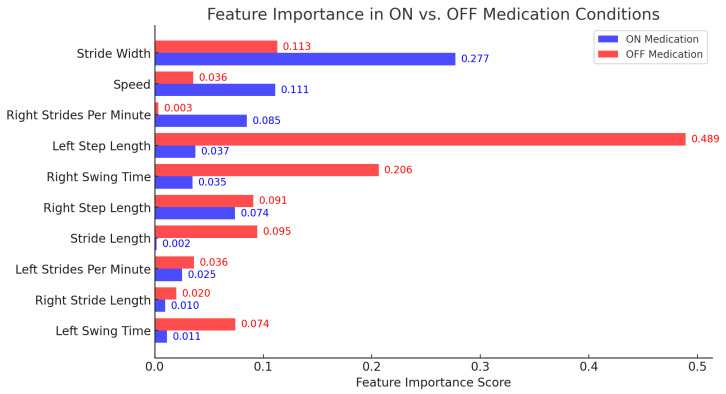
Feature importance comparison between ON and OFF medication conditions. The red bars indicate OFF medication, while blue bars indicate ON medication.

Feature importance analysis was conducted using a random forest regressor, incorporating both impurity-based feature importance and permutation-importance methods. This analysis allowed us to identify the most predictive gait parameters for FoG severity in each medication state, providing insight into how medication affects gait characteristics in PD patients (Figure 2).

In the ON medication condition, the most influential feature was stride width (0.27), indicating its strong association with gait stability under medication. Speed (0.11) and right strides per minute (0.08) were also key predictors, highlighting the role of gait velocity and cadence in FoG severity. Moderate contributors included left step length (0.03) and right swing time (0.03), suggesting that step asymmetry might be a relevant factor. Meanwhile, right step length (0.07) had a notable impact, whereas stride length (0.00) and right stride length (0.01) exhibited lower predictive power, indicating limited influence in the ON state.

In the OFF medication condition, the dominant predictor was left step length (0.48), reflecting the strong impact of step length variability on FoG severity when medication is absent. Right swing time (0.20) and stride width (0.11) followed, showing their importance in maintaining dynamic balance. Right step length (0.091) and right initial double limb support time (0.07) also contributed significantly, reinforcing the idea that postural transitions influence FoG episodes. Left swing time (0.03) and speed (0.03) were moderate predictors, while right steps per minute (0.00) and right terminal double limb support time (0.02) had minimal impact.

To categorize feature importance, a relative ranking approach was used. Features with importance values above 0.20 were considered highly predictive, as they accounted for a significant portion of the model’s explanatory power. Features between 0.05 and 0.20 were classified as moderate predictors, contributing meaningfully but not dominating the prediction. Features below 0.05 were deemed low importance, as their impact on FoG prediction was minimal [28].

These findings suggest that step length and stride width are among the most critical parameters for predicting FoG severity, with medication influencing their predictive relevance. Under medication, stride width and speed play a greater role, while in the absence of medication, step length variability and double limb support time become more prominent. These insights emphasize the importance of considering gait characteristics within specific medication states, offering potential guidance for targeted interventions and rehabilitation strategies for PD patients.

Figure 2 illustrates the feature importance scores for gait parameters in ON vs. OFF medication conditions. As seen, the importance of most features decreases when patients are ON medication.

### 4.3. Paired Evaluation of Medication Effects on Gait Patterns

To assess the effects of medication on gait characteristics in PD patients, a paired comparison of spatiotemporal gait parameters was conducted between ON and OFF medication states. The results provide insights into how medication influences gait dynamics, particularly in relation to step length, stance time, and gait speed (Table 3).

Step length and stride length: A significant reduction in step length and stride length was observed when patients were OFF medication. Specifically, left step length decreased from 0.53 m (ON) to 0.46 m (OFF) (*t* = 4.40, *p* = 0.00), and right step length followed a similar pattern, reducing from 0.54 m (ON) to 0.48 m (OFF) (*t* = 4.46, *p* = 0.00). Likewise, left stride length declined from 1.07 m (ON) to 0.94 m (OFF) (*t* = 4.50, *p* = 0.00), and right stride length decreased from 1.07 m (ON) to 0.94 m (OFF) (*t* = 4.63, *p* = 0.00). These findings suggest that medication plays a critical role in maintaining gait symmetry and step amplitude, reducing the characteristic shuffling gait often observed in PD patients.

Speed and temporal parameters: Gait speed was significantly lower in the OFF medication condition, dropping from 0.99 m/s (ON) to 0.84 m/s (OFF) (*t* = 5.15, *p* = 0.00). This suggests that medication facilitates a more dynamic and continuous walking pattern. Cycle time, which reflects the duration of a full gait cycle, was slightly longer in the OFF condition (1.12 s ON vs. 1.20 s OFF), although the difference was not statistically significant (*t* = −1.44, *p* = 0.16).

Stance and swing times: No significant differences were detected in swing time, with left swing time at 0.42 s (ON) vs. 0.41 s (OFF) (*t* = 1.13, *p* = 0.27) and right swing time remaining at 0.41 sec in both conditions (*t* = 0.09, *p* = 0.93). However, stance time was slightly prolonged in the OFF state, indicating greater time spent in the support phase, likely compensating for instability.

Double limb support and balance indicators: Although double limb support time was longer in the OFF condition (0.29 s ON vs. 0.38 s OFF), the difference was not statistically significant (*t* = −1.69, *p* = 0.11). Similarly, right initial double limb support time and right terminal double limb support time were both prolonged when OFF medication, indicating increased reliance on double support for stability.

## 5. Discussion

This study explored the role of spatiotemporal gait parameters in predicting FoG in PD, emphasizing the differences between ON and OFF medication states. The performance analysis of the random forest regressor revealed a stronger predictive capability in the OFF medication condition compared to the ON condition, highlighting the impact of dopaminergic treatment on gait dynamics and FoG severity prediction.

### 5.1. The Effect of Dopaminergic Medication on FoG Prediction

The results demonstrated that FoG prediction was more accurate in the OFF medication condition, where gait impairments appeared more distinct and stable. In contrast, in the ON condition, increased gait variability and compensatory strategies reduced the model’s predictive accuracy. The disparity in FoG prediction accuracy aligns with the levodopa paradox, where dopaminergic therapy alleviates many motor symptoms but does not consistently improve FoG [1,5]. The model’s higher predictive accuracy in the OFF state suggests that FoG in this condition is primarily driven by biomechanical disruptions—such as step length asymmetry, stride variability, and prolonged double limb support time—which remain relatively stable and predictable. In contrast, when patients were on medication, the model’s accuracy dropped, indicating that nearly half of the variability in FoG severity could not be explained by spatiotemporal gait parameters alone.

One possible explanation for this reduced predictive accuracy in the ON state is that dopaminergic medication reduces the likelihood and severity of FoG episodes, making it harder for the model to detect clear patterns. Since medication generally improves gait performance [11], FoG may have become less frequent or less pronounced, weakening the statistical relationship between gait parameters and FoG severity. This interpretation aligns with Scully et al. (2023) [29], who found that the FoG Severity Tool was less effective in detecting FoG during the ON state, suggesting that medication creates a floor effect, where FoG symptoms become harder to quantify. Additionally, Pérez-Lloret et al. (2014) [30] distinguished between “OFF freezing”, which improves with medication, and “ON freezing”, which remains resistant to treatment, reinforcing the idea that different mechanisms underlie FoG depending on medication status.

Rather than eliminating FoG, dopaminergic therapy may modify its presentation by influencing specific gait characteristics. In this study, right step length was significantly longer in the ON state (*p* < 0.001), suggesting that patients adjust their gait mechanics in response to medication. This aligns with prior findings indicating that dopaminergic treatment does not completely eliminate FoG but instead alters movement strategies, which may create individual variations in gait adaptation. Since FoG arises from dysfunction in the basal ganglia and frontal cortex, dopaminergic therapy may not fully restore these circuits, leading to medication-induced gait changes rather than complete symptom resolution. This is further supported by Jansen et al. (2023) [31], who found that long-term dopaminergic treatment may actually contribute to the persistence of FoG, even in patients who are already medicated. In other words, while medication improves general motor function, it does not guarantee the elimination of FoG, and its effects on gait patterns can vary between individuals, creating unpredictable influences on movement control. Since FoG is primarily caused by neurological dysfunction in the basal ganglia and frontal cortex, which play a crucial role in motor control, it is possible that dopaminergic medication does not fully restore these impaired neural circuits [32].

Another factor contributing to the lower predictive accuracy in the ON state is the increased gait variability introduced by the medication. Dopaminergic therapy affects motor control mechanisms, potentially altering how patients regulate stride, step initiation, and weight shifting. Interestingly, stride width did not significantly differ between ON and OFF states, contradicting previous assumptions that medication-induced balance improvements would play a key role in reducing FoG risk. These findings suggest that spatiotemporal gait parameters alone may not fully explain FoG in medicated patients, highlighting the need to incorporate cognitive, sensory, and neurophysiological factors into predictive models. In particular, 48% of the variability in FoG severity in the ON condition remained unexplained, indicating that nonmotor influences—such as cognitive load, attention control, anxiety, or medication fluctuations—may play a more significant role in FoG under dopaminergic therapy. This could explain why some PD patients continue to experience FoG despite medication, while others exhibit more unpredictable gait patterns that are harder to model using traditional spatiotemporal predictors.

These findings emphasize the need for a multi-dimensional approach to FoG prediction, especially in the ON state, where spatiotemporal gait parameters alone may not be sufficient. Future predictive models should integrate neurological, cognitive, and pharmacological factors to improve precision and better capture the complex interaction between medication, movement control, and FoG episodes. Understanding these mechanisms can also inform rehabilitation strategies, allowing clinicians to develop personalized interventions based on individual medication responses and gait characteristics.

In summary, while dopaminergic treatment improves some aspects of gait, it also introduces new challenges in FoG prediction and management. Future research should focus on understanding how medication modifies gait patterns and FoG severity, ultimately leading to more effective assessment tools and intervention strategies tailored to the needs of PD patients.

### 5.2. Feature Importance in FoG Prediction and Pairwise Comparisons: Identifying Key Gait Parameters

The model predicted FoG severity more accurately in the OFF state (R² = 0.82) than in the ON state (R² = 0.52), suggesting that FoG-related gait disturbances are more distinct without medication. In contrast, dopaminergic treatment introduced gait variability and compensatory mechanisms, reducing the predictive power of gait parameters and making FoG episodes less biomechanically distinct [33,34]. To further explore these effects, a feature importance analysis was conducted to identify the key gait predictors in each medication state.

#### 5.2.1. Key Predictors in the OFF Condition

In the OFF medication condition, left step length (0.48) emerged as the most influential feature, reinforcing its role in step length asymmetry and overall gait instability, which are well-established markers of FoG severity [6]. Additionally, right swing time (0.20) and stride width (0.11) were among the top predictors, suggesting that balance and swing phase duration significantly contribute to FoG severity in unmedicated patients. The importance of right step length (0.09) and right initial double limb support time (0.07) further highlights the role of postural transitions and weight-shifting difficulties in unmedicated patients.

#### 5.2.2. Key Predictors in the ON Condition

Conversely, in the ON medication condition, stride width (0.27) became the most significant predictor, suggesting that medication facilitates a broader stance as a compensatory mechanism to maintain balance [35]. This shift indicates that patients may rely more on lateral stability adjustments rather than step length regulation when medicated, potentially as an alternative strategy to counteract instability. Additionally, speed (0.11) and right strides per minute (0.08) were prominent features, reflecting how dopaminergic treatment affects cadence and gait velocity rather than simply restoring natural gait patterns. Notably, left step length (0.03) and right swing time (0.03) had a lower predictive impact, suggesting that step asymmetry plays a diminished role under medication. This implies that medication may reduce step length irregularities while shifting gait adjustments towards stride width and cadence-based compensation.

#### 5.2.3. Paired Comparisons of Gait Parameters

Paired comparisons of gait parameters between the ON and OFF medication states further illustrate the differential effects of dopaminergic treatment on movement control. The significant reduction in step length and stride length in the OFF state highlights the progressive gait impairments in unmedicated patients, reinforcing their role as key markers of FoG severity. Additionally, the observed decline in gait speed in the OFF state suggests an overall reduction in movement efficiency, potentially contributing to an increased risk of FoG episodes. However, the lack of significant changes in cycle time implies that medication does not strongly influence the overall gait cycle structure, but rather selectively modulates specific spatial and temporal parameters. The prolonged double limb support time in the OFF state reflects increased postural instability and a greater reliance on compensatory balance strategies, further confirming that weight-shifting difficulties are a hallmark of FoG in unmedicated patients.

Several gait parameters showed no significant differences between ON and OFF states, suggesting medication does not uniformly affect gait. Stance time (left: *p* = 0.11, right: *p* = 0.15), swing time (left: *p* = 0.27, right: *p* = 0.93), and stride width (*p* = 0.98) remained stable, possibly due to compensatory mechanisms. Step frequency metrics, including steps per minute (*p* > 0.20), also showed no significant changes, indicating cadence remains unaffected. Similarly, cycle time (*p* = 0.16) and double limb support time (*p* = 0.11) suggest medication reduces variability rather than altering gait mechanics. These findings highlight the complexity of medication effects, with step length and speed changing while other parameters remain stable, emphasizing the need for a comprehensive approach to gait adaptation in PD.

Overall, these findings indicate that medication does not simply optimize gait but rather modulates gait patterns in complex ways. While dopaminergic therapy enhances certain aspects of movement, such as stride length and gait speed, it also introduces compensatory strategies, such as lateral stability adjustments, that influence gait predictability. These findings reinforce the need for individualized medication strategies tailored to the preservation of gait in PD, ensuring that interventions account for both improvements in mobility and medication-induced gait variability.

### 5.3. Clinical Implications, Limitations, and Future Directions

The findings of this study have significant implications for clinical practice. By identifying specific gait parameters that predict the severity of FoG, clinicians can develop more customized rehabilitation strategies for patients with PD. For example, focusing on improving the mean length of left steps in patients experiencing FoG in the OFF state can improve mobility and reduce the risk of falling. Furthermore, understanding how medication alters gait dynamics can inform treatment plans, allowing more personalized approaches that consider individual patient responses to dopaminergic therapy. This could lead to the development of targeted interventions, such as cueing strategies or gait training programs, that address specific gait impairments associated with FoG

Despite promising findings, this study has several limitations. The sample size of twenty two PD patients may limit the generalizability of the results, as larger and more diverse cohorts are needed to validate the predictive models. Additionally, the study’s reliance on specific spatiotemporal gait parameters may overlook other relevant factors, such as dynamic characteristics, cognitive load, or environmental influences that could impact FoG prediction. Furthermore, the cross-sectional design does not allow the assessment of changes in gait dynamics over time, which could provide valuable insights into the progression of PD and FoG. Nonetheless, we recognize that a subject-wise split, where entire participants are assigned exclusively to either the training or testing set, could provide a more rigorous assessment of the model’s generalization, even with the random forest method, which does not store individual-specific information. Comparing this approach to our current method would be a valuable direction for future research. Finally, the study’s wide age range raises concerns about its impact on gait. However, accounting for age-related adaptations was not within our study’s scope. Future work should integrate age and disease duration to improve predictive accuracy.

Future studies should aim to expand on these findings by incorporating larger and more diverse patient populations to improve the generalizability of the results. Longitudinal studies could provide insight into how gait parameters change over time and their relationship with FoG progression. Furthermore, exploring the integration of multimodal sensor data, including cognitive assessments and environmental factors, could improve the accuracy of FoG prediction models. Investigating the effects of different dopaminergic treatments on gait dynamics can also yield valuable information to optimize therapeutic strategies for patients with PD.

## 6. Conclusions

This study demonstrated that spatiotemporal gait parameters can predict FoG severity in PD, with their predictive power varying between ON and OFF medication states. The findings showed that FoG prediction was more accurate in the OFF condition, where gait impairments were more distinct and stable. In contrast, in the ON medication state, greater gait variability and compensatory adjustments altered the predictive relationship between gait parameters and FoG, making detection more challenging.

The results revealed that while step length asymmetry and stride variability were key predictors in the OFF state, the ON condition showed a shift in gait adaptation, with medication influencing stride and step length rather than cadence-related parameters. This suggests that rather than simply reducing predictive power, medication alters which gait features contribute to FoG detection, emphasizing spatial gait characteristics over temporal ones. These findings highlight the need to account for medication-induced changes in gait dynamics when modeling FoG severity.

These findings highlight the need to account for medication-induced gait variability when developing predictive models and intervention strategies for FoG. Understanding how medication shifts the dominant gait predictors of FoG severity can help refine assessment tools and guide personalized rehabilitation approaches. Future studies should investigate the longitudinal effects of medication on gait variability and explore whether integrating cognitive or sensory factors could improve the robustness of FoG prediction models.

## Figures and Tables

**Table 1:** Summary statistics for age, height, weight, and BMI.

	Mean	Standard Deviation
Age	64.23	9.75
Height (cm)	166.02	6.96
Weight (kg)	71.36	12.46
BMI	25.84	3.87

**Table 2:** Performance metrics for OFF and ON medication conditions.

Metric	OFF Medication	ON Medication
Mean absolute error (MAE)	2.25	4.16
Mean squared error (MSE)	15.23	42.00
R^2^ Score	0.82	0.52

**Table 3:** Statistical comparison of spatiotemporal gait parameters between ON and OFF medication conditions. N is the number of gait cycles recorded while the patient was ON or OFF medication.

Variable	N	ON Mean	ON Std	OFF Mean	OFF Std	t-Value	*p*-Value
Left stance time (s)	22	0.70	0.14	0.79	0.36	−1.65	0.11
Left swing time (s)	22	0.42	0.03	0.41	0.04	1.13	0.27
Left step length (m)	22	0.53	0.13	0.46	0.16	4.40	0.00
Left steps per minute	22	107.61	11.64	105.37	17.55	1.07	0.30
Left stride length (m)	22	1.07	0.25	0.94	0.30	4.50	0.00
Left strides per minute	22	54.44	6.10	52.79	9.30	1.27	0.22
Right stance time (s)	22	0.71	0.14	0.79	0.36	−1.50	0.15
Right swing time (s)	22	0.41	0.04	0.41	0.05	0.09	0.93
Right step length (m)	22	0.54	0.13	0.48	0.14	4.46	0.00
Right steps per minute	22	110.44	13.09	106.52	20.21	1.23	0.23
Right stride length (m)	22	1.07	0.25	0.94	0.30	4.63	0.00
Right strides per minute	22	54.51	6.06	52.92	9.38	1.21	0.24
Speed (m/s)	22	0.99	0.28	0.84	0.31	5.15	0.00
Stride length (m)	22	1.07	0.25	0.94	0.30	4.58	0.00
Stride width (m)	22	0.10	0.04	0.10	0.03	−0.02	0.98
Cycle time (s)	22	1.12	0.15	1.20	0.35	−1.44	0.16
Double limb support time (s)	22	0.29	0.13	0.38	0.37	−1.69	0.11
Right initial double limb support time (s)	22	0.15	0.07	0.19	0.17	−1.83	0.08
Right terminal double limb support time (s)	22	0.14	0.07	0.19	0.21	−1.56	0.13
